# RETRACTION: Molecular Mechanism of Traditional Chinese Ointment of Xuzhou Qufu Shengji in Infected Wounds

**DOI:** 10.1155/ecam/9863575

**Published:** 2026-03-23

**Authors:** Evidence-Based Complementary and Alternative Medicine

RETRACTION: J. Shen, C. Li, L. Zhang, Y. Sun, L. Zhang, and L. Yang, “Molecular Mechanism of Traditional Chinese Ointment of Xuzhou Qufu Shengji in Infected Wounds,” *Evidence-Based Complementary and Alternative Medicine*, 2022, 4116563, https://doi.org/10.1155/2022/4116563.

The above article, published online on 12 January 2022 in Wiley Online Library (https://wileyonlinelibrary.com), has been retracted by John Wiley & Sons Ltd. The retraction has been agreed due to concerns identified in Figure 6, raised both directly and on PubPeer [1]. Specifically,

• There are unexpected, repeated regions between the 21d Vaselin and QFSJO panels.
• There are unexpected, repeated regions between the 14d QFSJO and Erythromycin panels.

After assessment of the concerns and author’s original images, the results and conclusions presented are deemed unreliable, and the article is retracted.

The authors did not respond when informed of the retraction.
